# Royal jelly enhances antigen‐specific mucosal IgA response

**DOI:** 10.1002/fsn3.29

**Published:** 2013-03-06

**Authors:** Hikaru Kai, Yuji Motomura, Shiro Saito, Ken Hashimoto, Tomoki Tatefuji, Nobutoki Takamune, Shogo Misumi

**Affiliations:** ^1^ Department of Pharmaceutical Biochemistry, Faculty of Medical and Pharmaceutical Sciences Kumamoto University Kumamoto Japan; ^2^ Institute for Bee Products and Health Science Yamada Apiculture Center, Inc. Okayama Japan

**Keywords:** Functional food, IgA, M cell, mucosal immunity, royal jelly

## Abstract

The effective uptake of antigens (Ags) by specialized M cells of gut‐associated lymphoid tissues is an important step in inducing an efficient intestinal mucosal immune response. In this study, royal jelly (RJ) was found to stimulate the differentiation of M‐like cells from human Caco‐2 cells in an in vitro M cell model. Furthermore, RJ and protease‐treated royal jelly (pRJ) efficiently enhanced transcytosis of FluoSpheres^®^ carboxylate‐modified microspheres from the apical side to the basolateral side in the model. Therefore, we evaluated the ability of pRJ to induce efficient mucosal immune responses in an in vivo nonhuman primate. Continuous oral administration of commercially available pRJ resulted in a significant enhacement of the antigen‐specific IgA response in stool sample. Interestingly, Caco‐2 monolayer assay demonstrated that ether extracts from pRJ efficiently increased the expression level of a universal M cell marker, glycoprotein 2 (gp2). These findings suggest that pRJ exhibits mucosal immunomodulatory properties via stimulation of effective uptake of Ags through M cells.

## Introduction

M cells in Peyer's patches (PPs) are instrumental in initiating mucosal immunity against antigens (Ags) invading across epithelial barriers (Kraehenbuhl and Neutra 2000). Although the intestinal tract is in direct contact with the external environment and is continuously exposed to large numbers of Ags, M cells mainly transcytose the Ags and deliver them to the underlying organized lymphoid follicles (Sansonetti and Phalipon 1999). Therefore, an intentional increase in the number of M cells in PPs or the enhancement of efficient transport of luminal Ags has a possibility of inducing the effective mucosal immunity against the Ags.

Current efforts to develop effective mucosal vaccines are mainly directed toward finding more efficient means of delivering appropriate Ags to the mucosal immune system and toward developing effective and safe mucosal adjuvants (Holmgren et al. 2003), because it has often been proved difficult to stimulate strong mucosal IgA immune responses and protection against pathogens by mucosal administration of Ags without Ags delivery and adjuvant systems. Furthermore, the high transcytotic ability of M cells makes Ags an attractive target for mucosally delivered vaccines because mucus secretion may flush out an applied mucosal vaccine at the mucosal site. Some studies showed that mucosal vaccine delivery can be improved using appropriate M‐cell targeting molecules such as lectins, its peptide mimetics and antibodies (Abs) specific to a fucose moiety presented at the surface of mouse (but not human) M cells (Wang et al. 2005; Nochi et al. 2007; Misumi et al. 2009; Mishra et al. 2011). However, challenges still remain, such as the identification of M cell target receptors that will reliably work in humans, and the safe reservation of M‐cell targeting molecules in humans. Therefore, inducing the increase in the number of M cells in human PPs or the enhancement of efficient transport of luminal Ags using functional foods or supplements that are taken orally in daily life may provide an alternative strategy to induce an effective Ags‐specifc mucosal immunity.

Royal jelly is a functional healthy food that possesses several health‐promoting properties. Previous studies demonstrated that RJ possesses numerous functional properties such as antiallergic (Okamoto et al. 2003), anti‐inflammatory (Kohno et al. 2004) and immunomodulatory properties (Sver et al. 1996; Majtan et al. 2006). These studies demonstrated both the immunostimulatory and immunosuppressive effects of RJ, which could be due to the presence of components possessing different immunomodulatory activities. However, almost nothing is known about the effect of RJ on the mucosal immune system. Therefore, we studied the effect of RJ on the mucosal immune system using an in vitro M cell model and an in vivo nonhuman primate model.

**Figure 1:**
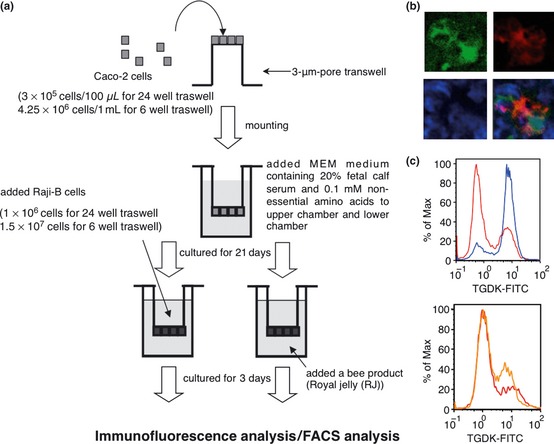
Royal jelly (RJ) promotes M‐like cell differentiation. (a) Outline of in vitro M cell model. (b) The Caco‐2/Raji B monolayer was stained with FITC‐labeled tetragalloyl‐D‐lysine dendrimer (TGDK) (green) or an anti‐gp2 antibody (red). The cell nucleus is shown in blue (DAPI staining). The pattern of triple fluorostaining of TGDK/gp2/4′, 6‐diamidino‐2‐phenylindole (DAPI) is shown. (c) Upper panel, M‐like cells differentiated from Caco‐2 cells (red line) after the Caco‐2 cells were cocultured with Raji B cells were TGDK‐positive cells (blue line). Lower panel, the treatment with RJ (orange) significantly increased the staining intensity in TGDK‐positive cells but that of DMSO (red) did not.

First, we evaluated whether RJ stimulates the differentiation of M‐like cells from human intestinal epithelial cells (Caco‐2 cells) in the in vitro M cell model in accordance with a slight modification of the method of Kernéis et al. (1997). As shown in Figure 1a, Caco‐2 cells that formed a complete monolayer after 21 days in culture acquire M cell‐like characteristics after being cocultured with Raji B cells for 3 days. As shown in Misumi et al. (2009), immunofluorescence analysis demonstrated that M‐like cells in the model showed costaining of an FITC‐labeled tetragalloyl‐d‐lysine dendrimer (TGDK) (green) and an anti‐gp2 antibody (red) (Fig. 1b), suggesting that TGDK specifically bound to human M‐like cells. On the other hand, RJ instead of Raji B cells was incubated on the apical side of the Caco‐2 monolayer for 3 days (Fig. 1a). RJ was supplied by the Yamada Apiculture Center (Okayama, Japan). RJ (8.5 mg) was dissolved with dimethyl sulfoxide (DMSO) and was further diluted in minimum essential medium (MEM) at a final DMSO concentration of 0.1%. As expected, FACS analysis showed an increase in the fluorescence intensity in TGDK‐positive cells after 3 days of coculture of the Caco‐2 monolayer with Raji B cells (Fig. 1c, upper panel). Interestingly, the treatment with RJ significantly increased in the fluorescence intensity in TGDK‐positive cells, although the intensity was lower than that in the coculture with Raji B cells (Fig. 1c, lower panel). These findings suggest that RJ stimulates the differentiation of M‐like cells from human Caco‐2 cells.

**Figure 2:**
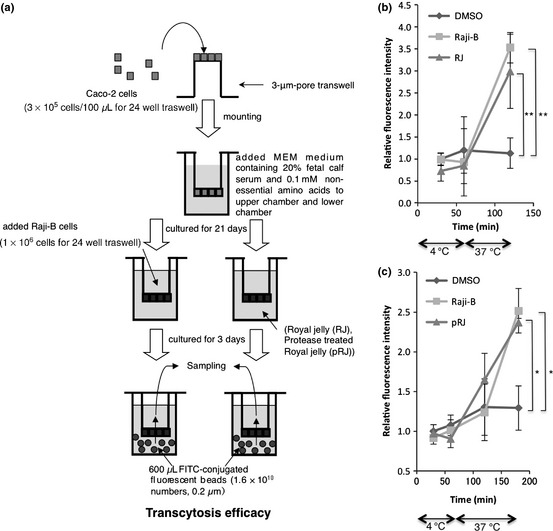
Both RJ and protease‐treated royal jelly (pRJ) promote antigen transcytosis across M‐like cells. (a) Outline of transcytosis assay in the in vitro M cell model. (b) To investigate the effect of RJ (B) or pRJ (c) on transcytosis activity in in vitro M cell model, the monolayers including M‐like cells induced to differentiate by Raji B cells, RJ, or pRJ as well as Caco‐2 control monolayers treated with DMSO as a control experiment were incubated with FluoSpheres^®^ carboxylate‐modified microspheres. The significance of difference (nonrepeated measures ANOVA) is indicated as follows: **, *P* < 0.01; *, *P* < 0.05. The *error bars* denote the standard deviation.

Transcytotic activity was also monitored, as shown in Figure 2a, in accordance with the method described in Misumi et al. (2009). The monolayers including M‐like cells or Caco‐2 control monolayers were incubated with FluoSpheres^®^ carboxylate‐modified microspheres (Life Technologies Corporation, Grand Island, NY). The microspheres that are transported from the lower chamber to the upper chamber were quantified using a CORONA multimicroplate reader. As shown in Figure 2b, the microspheres were efficiently transported at 37°C through the monolayers containing M‐like cells induced to differentiate by Raji B cells or RJ, but not through control monolayers treated with DMSO (**, *P* < 0.01 in nonrepeated measures ANOVA). Furthermore, we examined pRJ‐mediated transcytotic activity. pRJ was developed by the Yamada Apiculture Center to avoid RJ‐dependent anaphylaxis. For preparation of pRJ, RJ was digested with bacterial alkaline proteases, purchased from Kaken Pharmaceutical Co. Ltd., Japan, at temperature between 40°C and 50°C and at a pH between 7.8 and 9.0 for 4 h. After the enzymatic hydrolysis, proteases were denatured by heating the reaction mixture at 80°C for 15 min and then the mixture was lyophilized. As shown in Figure 2c, the microspheres were also efficiently transported through the monolayers containing M‐like cells induced to differentiate by pRJ (*, *P *<* *0.05 in non‐repeated measures ANOVA). These findings suggest that both RJ and pRJ induce an effective Ags‐specific mucosal immunity because they enhance the efficient Ags transport.

**Figure 3:**
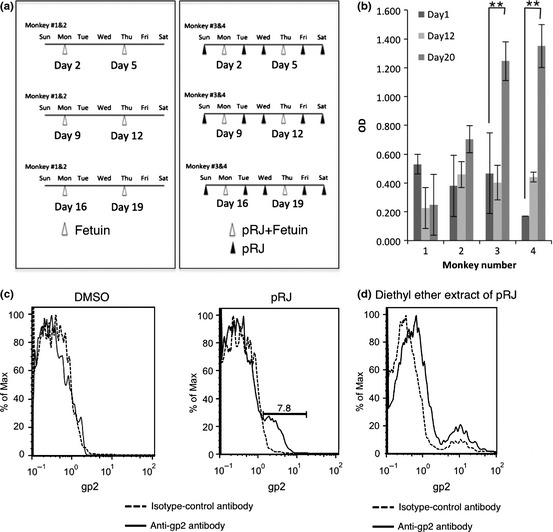
Effect of protease‐treated royal jelly (pRJ) on antigen‐specific mucosal IgA response and effect of pRJ or its diethyl ether extract on induction of gp2 expression. (a) Immunization schedule for cynomolgus macaques. Two cynomolgus macaques (Nos. 3 and 4) were orally immunized on days 2, 5, 9, 12, 16, and 19 with fetuin (1 mg) with pRJ (240 mg). Another two macaques (Nos. 1 and 2) were orally immunized with only fetuin on days 2, 5, 9, 12, 16, and 19. Stool sampling was performed on days 1, 12, and 20. This study (permission no. B24‐241) was approved and conducted in accordance with the guidelines of the Animal Care and Use Committee of Kumamoto University. (b) Stool samples (1/100 dilution) were examined to investigate whether anti‐fetuin mucosal IgA level can be raised in cynomolgus macaques by anti‐fetuin ELISA. Significance (repeated measures ANOVA) is indicated as follows: **, *P* < 0.01. The *error bars* denote the standard deviation. (c) To examine the RJ activity of pRJ that enhances gp2 expression in the in vitro Caco‐2 monolayer, the Caco‐2 monolayer treated with DMSO or pRJ (0.85 mg/mL) was subjected to flow cytometry using the anti‐gp2 antibody. (d) To investigate the effect of the diethyl ether extract of pRJ on the induction of gp2 expression, the Caco‐2 monolayer treated with the diethyl ether extract of pRJ (0.85 mg/mL) was subjected to flow cytometry using the anti‐gp2 antibody.

To examine the in vivo effect of pRJ on the ability to induce efficient mucosal immune responses in nonhuman primates, two cynomolgus macaques (male, 4 and 5 years old) were immunized with enteric‐coated capsules including fetuin (1 mg) with pRJ (240 mg) by oral administration according to the time schedule shown in Figure 3a. Another two rhesus macaques were immunized with enteric‐coated capsules including only fetuin (1 mg). Abs against fetuin in stool samples were detected by enzyme‐linked immunosorbent assay (ELISA). For evaluation of stool Abs, fecal pellets were resuspended in a sample buffer (0.1% sodium azide, 1 mmol/L EDTA, 0.05% Tween 20, 5% nonfat skim milk, and 1 mmol/L phenylmethylsulfonyl fluoride (PMSF), fecal weight:sample buffer = 1:4). The suspensions were centrifuged at 13,000*g* for 5 min to remove fecal solids. The processed fecal samples were subjected to ELISA of anti‐fetuin Abs. For ELISA, each well of a flat‐bottom 96‐well maxisorp microplate (Nunc, Thermo Fisher Scientific K.K., Tokyo, Japan) was coated with 50 μL of a coating buffer (pH 8.0, 50 mmol/L Tris‐HCl, 10 mmol/L MgCl_2_, 0.1% Tween 80) containing fetuin (100 μg/mL) and incubated at room temperature (RT) overnight. The wells were washed with 150 μL of Milli‐Q water with 0.1% Tween 80 with complete decanting and rinsed with Milli‐Q water. Subsequently, 200 μL of a blocking buffer composed of 1% skim milk in Milli‐Q water was added to each well, and incubated at RT overnight to occupy all unbound sites. Washing was repeated, as described above, followed by the addition of 50 μL of a fecal sample diluted at 1/100 in phosphate buffered saline(−) to each well. Plates were incubated for 1.5 h at RT and then washed with 0.1% Tween 80, and 50 μL of peroxidase‐conjugated goat anti‐monkey IgA (diluted at 1/10,000) was added to each well. The plates were incubated for 0.75 h at RT before washing them. 3, 3′, 5, 5′‐Tetramethylbenzidine solution (50 μL, Wako Pure Chemical Industries, Ltd., Osaka, Japan) as the substrate was added to each well and incubated at RT. Absorbance was measured at 450/630 nm using a microplate reader. Interestingly, fetuin‐specific IgA in stool samples was significantly induced in the group immunized with fetuin with pRJ on day 20 (Fig. 3b; **, *P* < 0.01 in repeated measures ANOVA). In contrast, the immunization of rhesus macaques with only fetuin did not effectively induce fetuin‐specific IgA in stool samples (Fig. 3b). This findings suggests that continuous oral administration of commercially available pRJ may be required to obtain a significant enhancement of the fetuin‐specific IgA response in stool samples.

Because the bioactive component of pRJ, which modulates the effective mucosal immunity, has not been fully characterized, we further examined the activity of the pRJ component that enhances the gp2 expression by in vitro Caco‐2 monolayer assay. The Caco‐2 monolayer was treated with MEM containing pRJ (0.85 mg/mL; final DMSO concentration, 0.1%) for 3 days. For FACS analysis, the resulting cells were stained with a rabbit anti‐gp2 antibody (IMGENEX Corporation, San Diego, CA) for 30 min on ice and then an Alexa488‐labeled anti‐rabbit antibody was used as the secondary antibody for staining. After 30 min of incubation on ice, the cells were washed and then analyzed using an EPICS XL flow cytometer (Beckman Coulter K.K., Tokyo, Japan). As shown in Figure 3c, the treatment with pRJ enhanced the expression of gp2. Because both RJ and pRJ could enhance transcytosis, the effect was exerted by the protease‐resistant components. Therefore, we examined whether the diethyl ether extract of pRJ could enhance gp2 expression. Interestingly, the diethyl ether extract of pRJ more efficiently enhanced gp2 expression. Although the relation between this extract of pRJ and a 57 kDa protein (royalactin) in RJ for queen differentiation (Kamakura 2011) have remained unknown, our findings suggest that the diethyl ether extract of pRJ contains a mucosal immunomodulator stimulating the efficient uptake of Ags through M cells.

Together, our data point out that pRJ enhances antigen‐specific mucosal IgA responses by stimulating the efficient uptake of Ags through M cells and that the M cell‐mediated Ag sampling procedure is believed to be an essential and critical step for establishing a successful mucosal immune response.

## Conflict of Interest

None declared.
